# The Applications of Metabolic Glycoengineering

**DOI:** 10.3389/fcell.2022.840831

**Published:** 2022-02-17

**Authors:** Liwei Ying, Junxi Xu, Dawei Han, Qingguo Zhang, Zhenghua Hong

**Affiliations:** ^1^ Orthopedic Department, Taizhou Hospital Affiliated to Wenzhou Medical University, Linhai, China; ^2^ Enze Medical Research Center, Taizhou Hospital, Wenzhou Medical University, Linhai, China; ^3^ Department of Medical Oncology, The Second Affiliated Hospital, Zhejiang University School of Medicine, Hangzhou, China

**Keywords:** metabolic glycoengineering., cell-based therapy, cell membrane modification, ManNAc analogs, imaging

## Abstract

Mammalian cell membranes are decorated by the glycocalyx, which offer versatile means of generating biochemical signals. By manipulating the set of glycans displayed on cell surface, it is vital for gaining insight into the cellular behavior modulation and medical and biotechnological adhibition. Although genetic engineering is proven to be an effective approach for cell surface modification, the technique is only suitable for natural and genetically encoded molecules. To circumvent these limitations, non-genetic approaches are developed for modifying cell surfaces with unnatural but functional groups. Here, we review latest development of metabolic glycoengineering (MGE), which enriches the chemical functions of the cell surface and is becoming an intriguing new tool for regenerative medicine and tissue engineering. Particular emphasis of this review is placed on discussing current applications and perspectives of MGE.

## Introduction

The cell surfaces are dominated by multiple functional molecules, which play essential roles in regulating intercellular communications, molecules selective transportation and intracellular associated signaling pathways (Abbina et al., 2018). Understanding and manipulating the cell surface have aroused crucial topics in fundamental researches on cell behaviors, novel biotechnical applications and therapeutic exploitation (Nischan and Kohler, 2016). Compared with other molecules on cell surface, glycans signify the function of a cell and specify how it interacts with its surroundings (Du and Yarema, 2010). Although there are abundant chemical groups on the cell surface, only a handful of functional groups can be used for direct covalent bond formation reactions under suitable conditions. Since cell membrane modification can be exploited to rapidly provide additional cell functionality, therefore, it is critical for endowing or improving cellular biological behaviors, including immuno-evasion, adhesion and homing.

Redecorating cell surfaces with genes encoding peptides or proteins using conventional genetic engineering is a well-established strategy, which has been extensively applied in basic research and translational medicine (Rao et al., 2020). For example, previous reports have shown that using surface-displayed genetically engineered chimeric antigen receptor (CAR) T cells to recognize cancer antigens for the treatment of acute lymphoblastic leukemia (Kuehn, 2017). Although quite practical, such traditional genetic manipulation approaches are limited to displaying natural genetic encoded molecules on cellular surface, but cannot display non-genetically encoded molecules, such as cytotoxic drugs, fluorophores, spectroscopic probes, affinity tags, and vaccine adjuvants (Plumet et al., 2021). In addition, viral vectors are associated with high risks of genetic integration which may cause tumorigenesis and stimulate immunogenic responses (Lee et al., 2018).

Metabolic glycoengineering (MGE) - a technique where monosaccharide analogs are introduced into the metabolic pathways of a cell and are biosynthetically incorporated into the glycocalyx on cell membrane - has been shown to be precious for engineering cellular surface properties to allow manipulation of cellular behaviour and function using natural or unnatural functional groups (Du et al., 2009; Nagasundaram et al., 2020). Over the last 2 decades, more than 20 unnatural mannosamines that are suitable for MGE, have been introduced. These N-acetyl d-mannosamine (ManNAc) analogs can be divided into two groups, namely aliphatic and bioorthogonal analogues (Wratil et al., 2016). MGE can equip cell surfaces with various unnatural functional groups, such as ketone-, azide-, thiol-, or alkyne-modified glycans, which can then be combined with abundant ligands via bioorthogonal chemoselective ligation reactions, thereby extremely boosting versatility of this strategy. Specifically, MGE has various advantages, they are: 1) stable under physiologic conditions; 2) applicable to all cell types; 3) innocuous to the modified cell; and 4) reversible upon administration of a controlled precursor (Csizmar et al., 2018). Currently, cell membrane glycoengineering is a powerful tool that has been widely applied across the following fields: 1) endowment of cells with new abilities (Wang et al., 2020); 2) glycoengineered membrane self-assembly servers as vehicles to coat nanoparticles (Rao et al., 2020); 3) *in vivo* cell tracking (Lim et al., 2019); 4) metabolic glycan labelling (Hu et al., 2018b; Lim et al., 2019; Costa et al., 2020; Wang and Mooney, 2020); 5) cell and drug delivery (Lee et al., 2018; Xia et al., 2020); and 6) single cell encapsulation (Kim et al., 2018; Oh et al., 2020).

**SCHEME 1:**
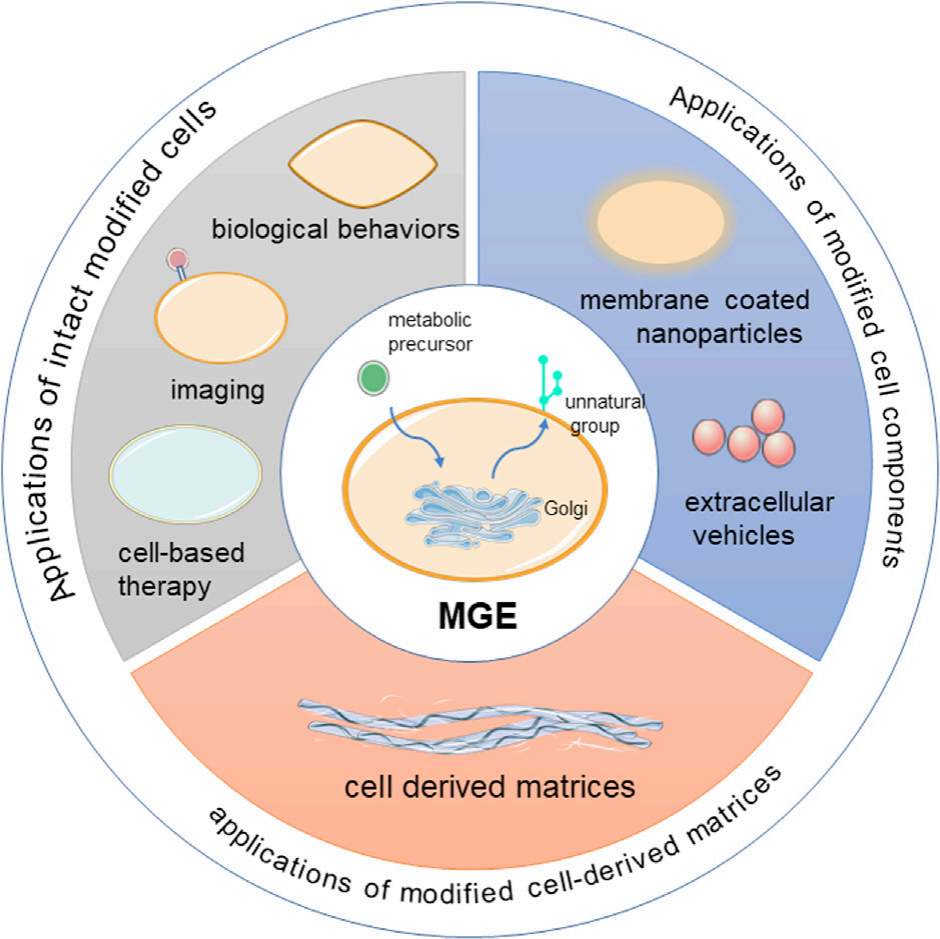
Overview of the applications of MGE in cells.

Currently, metabolic glycoengineering has numerous applications. If divided according to the cell structure, the applications can be divided into the following three broad types, namely the applications of intact cells, cell components and cell-derived matrices (Scheme 1). This review systematically summarizes current approaches and applications for cell surface glycoengineering, both *in vitro* and *in vivo*. We highlight key examples of MGE in each section, and provide perspectives and future trends of this rapidly growing field.

### Applications of Intact Modified Cells

#### Modulation of Cell Biological Behaviors

Previous evidences have shown that most cellular glycans on the outer surface of membrane mediate or modulate cell-cell, cell-molecule, and cell-matrix interactions, which are essential for the development and functioning of cells (Du and Yarema, 2010). Pretreatment of peracetylated N-thiolglycolyl-d-mannosamine (Ac5ManNTGc) resulted in expression of thiol on cell surface, which stimulated cell adhesion to complementary gold or maleimide-derivatized substrates (Sampathkumar et al., 2006). In addition, treatment of HL60-cells with N-propanoylmannosamine (ManNProp) increased cell adhesion to fibronectin by activating *β*1-integrin (Villavicencio-Lorini et al., 2002). Findings from other research have revealed significant improvement in adhesion ability of leukocytes, Jurkat cells, neural cells, mesenchymal stem cells (MSCs) and A549 cells after treatment with ManNAc analogs (Du et al., 2009; Du et al., 2011; Koo et al., 2015; Koo et al., 2016; Lo et al., 2016). However, Nagasundaram et al.(Nagasundaram et al., 2020) found a marked reduction in adhesion of MCF7 breast cancer cells to laminin upon treatment with different ManNAc analogs. In addition, they found that application of all non-natural sialic acid precursors downregulated N-acetylneuraminic acid (Neu5Ac) and polysialic acids (polySia), and further suppressed adhesion and migration.

MGE can also change the differentiation ability of cells. For example, supplement of human stem cells with Ac5ManNTGc was found to improve their differentiation towards a neural lineage (Sampathkumar et al., 2006). ManNProp reportedly promoted monocytic differentiation of HL60-cells (Horstkorte et al., 2004). Both Ac_5_ManNTProp and Ac_5_ManNTBut, which display thiol groups on the cell surface, were found to suppress adipogenic differentiation in hADSCs, but they did not interfere with differentiation to a glial lineage (Du et al., 2021). Moreover, both osteogenic and adipogenic differentiation were inhibited, when MSCs were pre- or continuous treated with 3F-Neu5Ac (Templeton et al., 2021).

MSCs incubated with ManNProp significantly upregulates sialyl Lewis X (sLeX) (Natunen et al., 2013), an epitope that promotes osteotropism (Dykstra et al., 2016; Sánchez-Martínez et al., 2021), augments neurotropism (Merzaban et al., 2015) and improves ischemia-reperfusion by increased homing efficacy towards porcine heart (Lo et al., 2016). In another study, Byeongtaek et al. (Oh et al., 2020) coated each neural progenitor cell (NPC) with a layer of polymer, via click-chemistry and glycoengineering. The cells enhanced trophic factor release by optimizing stiffness of the polymer coating, which reduced the required number of cells through augmenting the efficacy of each NPC. On the other hand, supplementation with 3F-Neu5Ac was found to improve adhesion and elevate the rate of migration of MSCs, thereby promoting their survival in an *in vitro* ischemia model (Templeton et al., 2021). In summary, MGE can effectively modulate cell biological behaviors (Table 1). Also, we conclude that the effects of MGE on cellular activities are dependent on cell types and different kinds of ManNAc analogs.

**TABLE 1:** The modulation of cell biological behaviors by MGE.

Cell biological behaviors	Cell type	Precursors	Groups on cell surface	Promotion (+) suppression (-)	References
Adhesion	human embryoid body–derived stem cells (hEBD)	Ac5ManNTGc	Thiols	Gold or maleimide +	Sampathkumar et al. (2006)
	HL60-cells	ManNProp	N-propanoyl	Fibronectin +	(Villavicencio-Lorini et al., 2002; Horstkorte et al., 2004)
	human Jurkat T-lymphoma derived cells (Jurkat cells)	Ac5ManNTGc	Thiols	Gold or maleimide +	Du et al. (2011)
	HL60 cells, HeLa and Jurkat cells	Ac5SiaC5F5	trifluorobutanoyl	Fibronectin -	Dafik et al. (2008)
	MCF7 breast cancer cells	ManNProp, ManNBut	N-propanoyl, N-butanoyl	Laminin -	Nagasundaram et al. (2020)
	MSCs	ManNPent, ManNHex	N-pentanoyl, N-hexanoyl	12-well plate + tumor cells +	Templeton et al. (2021)
	Macrophages	3F-Neu5Ac sgc8-SH	—	Tetrazine and transcyclooctene conjugation	Sugimoto and Iwasaki, (2018)
	Jurkat cells and A569 cells	Ac4ManNAz	Thiols	—	(Koo et al., 2015; Koo et al., 2016)
	NT2 neurons	ManNBut	Azide	Integrin-mediated +	Mahal et al. (2001)
	SW1990 pancreatic cancer cell	1,3,4-O-Bu3ManNAc	N-butanoyl sLe^X^		Almaraz et al. (2012)
Differentiation	hEBD	Ac5ManNTGc	Thiols	neuronal +	Sampathkumar et al. (2006)
		ManNProp	N-propanoyl	monocytic +	Horstkorte et al. (2004)
	HL60-cells hADSCs	Ac5ManNTProp and Ac5ManNTBut	Thiols	adipogenesis –	Du et al. (2021)
	MSCs	3F-Neu5Ac	—	glial lineage +–	Templeton et al. (2021)
	PC12 cerebellar neurons	ManNProp	N-propanoyl	osteogenesis and adipogenesis –	Kontou et al. (2008)
	hMSC	ManNProp	N-propanoyl	neuronal +	Schmidt et al. (1998)
		Ac4ManNAz	Azide	oligodendroglial +	Altmann et al. (2021)
				osteogenic and adipogenic +	
Migration	MSCs	3F-Neu5Ac	N-propanoyl, N-butanoyl	—	Templeton et al. (2021)
	MCF7 breast cancer cells	ManNProp, ManNBut, ManNPent, ManNHex	N-pentanoyl, N-hexanoyl	-	Nagasundaram et al. (2020)
Secretion	neural progenitor cell	Ac4ManNAz sgc8-SH	azide	+	Oh et al. (2020)
	macrophages		Thiols	+	Sugimoto and Iwasaki, (2018)
	PC12	ManNProp	N-propanoyl	+	Horstkorte et al. (2010)
Homing	MSCs	3F-Neu5Ac	/sLe^X^	+	Templeton et al. (2021)
	SW1990 pancreatic cancer cell	1,3,4-O-Bu3ManNAc		+	(Almaraz et al., 2012; Agatemor et al., 2019)
Survival	MSCs	3F-Neu5Ac	—	Ischemia +	Templeton et al. (2021)
	Macrophages	Ac4ManNAz	Azide	+	Mao et al. (2020)
	Jurkat cells	Ac5ManNTGc	Thiols	Gold or maleimide +	Du et al. (2011)

The cell biological behaviors were modulated by providing metabolic precursors. The migration of fluorescent labeled cells is introduced in *Imaging and Tracking*. Abbreviated: 2,4,7,8,9-pentaacetyl-3Fax-Neu5Ac-CO2Me (3F-Neu5Ac), N-butanoyl-d-mannosamine (ManNBut), N-pentanoyl mannosamine (ManNPent), N-hexanoyl mannosamine (ManNHex), thiol-terminated nucleic acid aptamers (sgc8-SH), *N*-azidoacetylmannosamine (Ac_4_ManNAz) and N-alkyneacetylmannosamine (Ac_4_ManNAl). Other abbreviations were not described in detail in the original text.

#### Imaging and Tracking

Various targetable chemical groups, such as azides (N_3_-), alkynes, thiols, and ketones can be successfully generated into cell glycans via MGE (Du et al., 2009). Imaging molecules, like fluorescence dyes or imaging agents, are then labeled with the generated chemical groups by click chemistry. Notably, N_3_-containing artificial chemical precursors are the most widely used, owing to the fact that these metabolites (N_3_-groups) can be specifically conjugated with various bioorthogonal agents, such as dibenzylcyclooctyne (DBCO) and bicyclo [6.1.0], nonyne (BCN) via bioorthogonal copper-free click chemistry under both *in vitro* and *in vivo* conditions. In addition, their inherent properties, including nontoxicity, high stability, and rapid reaction time under physiological conditions, make them highly suitable for labeling of live cells (Lim et al., 2019). To date, two ways of cell imaging or tracking *in vivo* have been described. The first involves direct injection of glycoengineered cells labeled with fluorescent dye (Seungho Lim et al., 2021), while the second entails intravenous (Lim et al., 2019), peritoneal (Prescher et al., 2004), or intratumoral (Koo et al., 2012) injection of precursors. After injection, the generated azide groups are able to bind DBCO/BCN group-modified nanoparticles *in vivo*. These nanoparticles can comprise fluorescence dyes, superparamagnetic iron oxide and so on. Apart from fluorescence, magnetic resonance imaging and computed tomography can also be used for cell imaging. Besides imaging tumors in living animals, cancer diagnosis is also the promising outlook for MGE, where it can be used for identification of biomarkers based on the capture and analysis of bioorthogonally modified glycoconjugates (Badr et al., 2017). Interestingly, Laughlin et al. (2008) achieved three-dimensional spatiotemporal imaging by treating zebrafish embryos with N-azidoacetylgalactosamine (Ac4GalNAz). The azide groups could only be generated on embryo surface that was newly grown and then conjugated different color of Alexa Fluor, which could be used to study embryo development in zebrafish. Overall, this indicates that unnatural groups generated exogenously can be used as target molecules, and can then be applied for imaging of live cells *in vivo* (Table 2).

**TABLE 2:** Applications of MGE for cellular imaging and tracking.

Methods	Cell type	Precursors	Groups on cell surface	Ligands	Location	References
Injection of modified cells	chondrocytes	Ac4ManNAz	Azide	DBCO-Cy650	hip joint	Yoon et al. (2016)
	ADSCs	Ac4ManNAz	Azide	DBCO-Cy5	inner thigh muscle	Lee et al. (2016)
	MSCs	Ac4ManNAz	Azide	BCN-Cy5.5 + Fe3O4	brain	Lim et al. (2019)
	MSCs	Ac4ManNAz	Azide	BCN-CNP-Cy5.5/IRON/GOLD	dorsal subcutaneous region	Lee et al. (2017b)
	cytotoxic T-cells	Ac4ManNAz	Azide	DBCO-Cy5.5	tumor	Kim et al. (2020)
	hEPCs	Ac4ManNAz	Azide	DBCO-Cy5	gracilis muscle	Seungho Lim et al. (2021)
Intratissue injection of metabolic precursor or glycoengineed cells	A549 tumor cells	Ac4ManNAz	Azide	DBCO-Cy5	tumor	Koo et al. (2012)
	4T1 cells	AzAcSA	Azide	BCN-TPET-TEG	tumor	Hu et al. (2018a)
	A549 cells	Ac4ManNAz	Azide	DBCO-Cy5	mouse liver	Kang et al. (2014)
	MCF-7 cells	Ac4ManNAz	Azide	DBCO-Cy5	tumor	Qiao et al. (2020)
Intravenous injection of metabolic precursor	tumor cells	Ac3ManNAz-PAMAM [G4]	Azide	ADIBO-Cy5.5	tumor	Lee et al. (2017a)
	MDA-MB-231 human breast cancer cells	cRGD−S-Ac3ManNAz	Azide	TPEBAI	tumor	Hu et al. (2018b)
	tumor cells	RR-S-c_3_ManNAz	Azide	DBCO-Cy5.5	tumor	Shim et al. (2016)
	MCF-7 cancer cells	Ac4ManNAz	Azide	DBCO-AIE dots	tumor	Zhang et al. (2019)
	—	LP-9AzSia	Azide		Mouse brain	Xie et al. (2016)
	tumor cells	Ac4ManNAz	Azide	DBCO-Cy5	tumor	Yujia Zhao et al. (2021)
	tumor cells and solid tumors	Ac4ManNAz-LP	Azide	DBCO-ZnPc-LP	tumor	Du et al. (2017)
	MCF-7 cancer cells	ZIF-8-Ac4GalNAz	Azide	DBCO-Cy5	tumor	Zhengwei Liu et al. (2021)
other	zebrafish embryo	Ac4GalNAz	Azide	DIFO–Alexa Fluor	zebrafish embryo	Laughlin et al. (2008)

The surface of cells could be labeled with bioorthogonal chemical groups by MGE, which can be further conjugated with fluorescence dyes or nanoparticles with imaging probes by click chemistry, *in vitro* and *in vivo*. Abbreviated: human embryonic stem cells-derived endothelial progenitor cells (hEPCs); a cathepsin B-specific cleavable peptide moiety (Lys-Gly-Arg-Arg, KGRR), a spacer linker of p-aminobenzyloxycarbonyl (S), and the metabolic precursor of triacetylated N-azidoacetyl-d-mannosamine (Ac3ManNAz), resulting in RR-S-Ac3ManNAz; the alkyne-functionalized water-soluble bioorthogonal turn-on probe (TPEBAI), T PEBAI with good water solubility and yield low fluorescence in aqueous media; azide-modified acetyl sialic acid (AzAcSA); nanomicelle of Ac4ManNAz (Ac4ManNAz-LP); aggregation-induced emission (AIE).

#### Cell-Based Therapy

Cell-based therapy plays a tremendous role in cancer immunotherapy, drug delivery, and tissue regeneration. Modification of cells, coupled with manipulation of their functions based on purposive therapeutic designs have generated numerous scientific interests in biomedical research. Cell-based therapy that alters cell biological activity via MGE has been discussed above (*Modulation of Cell Biological Behaviors*). This section discusses application of metabolic glycoengineered cells in cancer therapy.

Immunotherapy for cancer treatment is mainly constrained by lack of tumor specific antigens and immune tolerance. Manual introduction of chemical receptors onto the cell surface can well solve these drawbacks. Use of metabolic glycoengineered cells in the field of tumor therapy has been documented. Generally, target non-natural molecules on tumor cell membrane are generated using two approaches, namely intravenous administration (Lee et al., 2017a) and intratumoral injection (Koo et al., 2012) of metabolic precursors. In addition, several methods, such as generating a neoantigen (Yujia Zhao et al., 2021), chemo-photothermal therapy (Du et al., 2017), drug therapy (Layek et al., 2016) and synergistic therapy (Qiao et al., 2020), among others, are applied for tumor treatment.

Metabolic precursors accumulate in tumors usually through tumor-specific enhanced permeability and retention (EPR) effect after i. v. injection (Qiao et al., 2020). Although this method can easily introduce functional groups on the cell surface, it is still a great challenge to selectively label interested cell types *in vitro* and *in vivo*. Wang et al. (2017) took advantages of cancer-overexpressed enzymes to selectively cleave caged ether bonds converted by anomeric acetyl groups in tetraacetyl-N-azidoacetylmannosamine (Ac4ManAz) and found azide groups were introduced on the surface of cancer cells. On the other hand, Hu et al. (2018b) utilized over-expressed αvβ3 integrin on tumor cell membranes and developed a dual-responsive metabolic precursor, termed cRGD-S-Ac3ManNAz. Functionally, cRGD can be specifically recognized by αvβ3 integrin, while the disulfide group is responsive to intracellular glutathione. Both of the above methods can selectively generate unnatural glycans on cell membranes.

Apart from modifying tumor cells via MGE, glycoengineered immunocytes can also be used in tumor treatment. Particularly, T cells treated with Ac4GalNAz can introduce N_3_ on cell surface, thereby specific tumor targeting was initiated through a bio-orthogonal click reaction between N_3_ and BCN. This artificial targeting strategy has been shown to remarkably promote recognition and migration of T cells to tumor cells, thereby elevating cytotoxicity of T cells against different types of tumor cells by 2–4 times (Li et al., 2019). CAR T-cells neither express sLeX nor bind E-selectin. Results from a previous study showed that fucosylated human CAR-T cells upregulated sLeX expression upon exposure to type 2 sialylLacNAc, a precursor of sLeX. In fact, these fucosylated cells infiltrate the marrow at an efficiency that is 10-fold than that in unfucosylated cells (Mondal et al., 2019). Dendritic cell (DC) modification for enhancement of antigen presentation has emerged as a highly successful strategy in tumor immune therapy. Previous studies demonstrated that synthetic glycopolymer modified on the DC surface through facilitating DC interaction with T cells markedly elevated T cell activation and results in higher tumor toxicity (Yu et al., 2020).

Natural killer (NK) cells are cytotoxic cells with important functions in antitumor immunity, proposed as a promising alternative approaches to extend T cell-based therapy (Mehta and Rezvani, 2018). However, NK cells do not possess inherent targeting abilities towards cancer cells, and are also known to adversely affect endogenous gene uptake, a phenomenon that causes low transgene expression (Matosevic, 2018). CD22, a sialic acid binding protein, is lowly expressed on B cells. Previous studies showed that although antibody-based agents targeting CD22 on B lymphoma and leukemia cells were clinically efficacious against these malignancies, they also attacked normal B cells resulting in immune deficiency (Müller and Nitschke, 2014; Hong et al., 2020). Whereas, the abilities of glycoengineered of NK cells to bind and kill CD22 ^+^ lymphoma cells were significantly improved when cells were modified with 9-O modified sialic acid-based CD22 ligands (Haso et al., 2013; Ereño-Orbea et al., 2017). For example, NK cells were found to uptake metabolic precursors MPB-sia one and BPC-sia two and transform them into CD22 ligands through the cellular glycosylation machinery (Wang et al., 2020). Overall, these evidences (Table 3) indicate that cancer immunotherapy was potentiated by providing a simple and general metabolic glycoengineering-based cell therapy.

**TABLE 3:** Metabolic glycoengineered cells for cancer treatment.

Methods	Cell type	Precursors	Groups on cell surface	Ligands	Functions	References
The application of tumor cells	tumor cell	Ac4ManNAz	Azide	DBCO-Pam3CSK4	evoked both the humoral and the T-cell-dependent antitumor immune responses	Yujia Zhao et al. (2021)
	MCF-7 breast cancer cells	Ac4ManNAz	Azide	DLQ/DZ	drug delivery and cancer treatment	Qiao et al. (2020)
	tumor cell	DCL-AAM	Azide	DBCO–DOX	drug delivery and cancer treatment	Wang et al. (2017)
	MDA-MB-231 human breast cancer cells	cRGD−S-Ac3ManNAz	Azide	TPEBAI	selective cancer cell ablation	Hu et al. (2018b)
	HeLa and B16F10 cells	Ac4ManNAz	Azide	DBCO-RNase A	cleave RNA to kill cancer cells	Ziyin Zhao et al. (2021)
	pancreatic cancer cells	1,3,4-O-Bu3ManNAz	Azide	EGFR-targeting TKI drugs	restore sensitivity to erlotinib and gefitinib	Mathew et al. (2015)
	HepG2 cancer cell	GalAz	Azide	DBCO-DOX	cancer treatment	Wang et al. (2019)
	KB, HEK-293, and MCF7	Ac3ManNAz	Azide	DBCO--rhamnose (Rha)	recruit anti-Rha antibodies, leading to the destruction of target cells	Li et al. (2018)
	4T1 cells	Ac4ManNAz	Azide	Hf-AIE-PEG-DBCO	Radiodynamic therapy and radiotherapy under X-ray irradiation	Jingjing Liu et al. (2021)
	A549 and MCF-7 cell lines	Ac4ManNAz	Azide	GON-DBCO-DOX	drug delivery and cancer treatment	Meghani et al. (2018)
The application of other type of cells	MSCs	Ac4ManNAz	Azide	DBCO-paclitaxel	active tumor homing	Layek et al. (2016)
	DC	pMAM/pMAG	glycopolymers	mannose/glucose receptors on the T cell surface	promote the T cell activation by enhancing cell interactions between DC and T cell	Yu et al. (2020)
	NK cells	MPB-sia 1 and BPC-sia 2	CD22 ligands	CD22 ^+^ lymphoma cell lines	anticancer Immunotherapy	Wang et al. (2020)
	NK92MI cells	Ac4ManNAz	Azide-DBCO-7D12	EGFR on tumor cells	Promote the interaction between NK cells and tumor cells	Gong et al. (2021)
	Jurkat T cells	Ac4ManNAz	Azide-Tri-Adam	Azide-Tri-β-CD on A549 cells	the accumulation of Jurkat T cells at the surface of A549 cells activates NK cells	Plumet et al. (2021)
	T cells	Ac4GalNAz	Azide	BCN on tumor cells membrane	enhancing T cell recognition and cytotoxicity to tumor cell	Li et al. (2019)
	NK-92 cells	N3-SA	Azide	alkyne modified cetuximab	anticancer immune therapy	Xianwu Wang et al. (2021)

Abbreviated: DLQ/DZ, DBCO modified low molecular weight heparin-quercetin co-encapsulate DOX (doxorubicin) and ZnPc (zinc phthalocyanine); DCL-AAM, 1-((4-(2,6-diacetamidohexanamido)phenyl) (phenyl)methoxy)-3,4,6-triacetyl-N-azidoacetylmannosamine (histone deacetylase (HDAC)/cathepsin L (CTSL)-responsive acetylated azidomannosamine; gelatin-oleic nanoparticles (GON), synthesized poly-mannose (pMAM); poly-glucose (pMAG); tyrosine kinase inhibitor drugs (TKI); 9-azido N-acetyl neuraminic acid methyl ester (N3-SA).

### Applications of Modified Cell Components

Cell component modification is recently applied; thus, this approach is not as common as that of intact cells. In this section, we highlight the modification of cell components in two parts for the first time: extracellular vehicles (EVs) and cell membrane. EVs, endogenously secreted by cells, have bioactive components that correspond to unique biological functions (van den Boorn et al., 2013). Unfortunately, most of bare EVs are excreted by the reticuloendothelial system via the liver and spleen, when they are systemically administered (Smyth et al., 2015). Therefore, understanding biodistribution of EVs *in vivo*, coupled with their therapeutic efficacy and potential toxicity are imperative to their therapeutic application. Previous studies have shown that glycosylation of EVs can effectively solve the biodistribution of EVs (Williams et al., 2018). For example, Lim et al. decorated EV-secreting donor cells with an azide group via MGE using Ac4ManNAz and prepared DBCO-terminated PEGylated hyaluronic acid (DBCO-PHA) to specifically label the N_3_ group generated exogenously on the cells. PHA not only prolongs blood circulation, but also exhibits specific binding affinity to CD44 (Choi et al., 2011). Consequently, PHA-decorated EVs were found to accumulate in CD44-abundant tissues, such as rheumatoid arthritis and tumor (Gyeong Taek Lim et al., 2021). In addition, You et al. (2021) reported the fine surface editing of EVs by the MGE of ADSCs to target activated macrophages and promote M1-M2 polarization.

Cell membrane-coating has emerged as a promising nano-delivery system for drugs, owing to various ideal characteristics that include small size, safety, biocompatibility, biorecognition, high stability and target specificity (Zhu et al., 2016; Zhen et al., 2019). Consequently, previous reports have documented the wide application of genetically engineered cell-membrane-coated nanoparticles in cancer immunotherapy and drug delivery (Rao et al., 2020; Xia et al., 2020). Notably, a specific artificial targeting strategy *in vitro* and *in vivo* based on MGE-click chemistry was developed with the aim of addressing limitations associated with endogenous protein receptors dependent. For example, Hao Wang et al. (2021) encapsulated photosensitizer IR-780 into the N_3_-labeled cell membrane, by incubating cells with Ac4ManNAz, and endowed cells in psoriatic lesions with DBCO groups via subcutaneous injection with Ac4ManN-DBCO. The bioorthogonal click chemistry between the N_3_ and DBCO groups allowed efficient accumulation of IR-780 in lesion skin, thereby promoting photodynamic and photothermal therapy. On the other hand, Xiao et al. (2021) coated an N_3_-labeled DC membrane on imiquimod-loaded polymeric nanoparticles, and sequentially modified anti-CD3ε antibody via click chemistry. The nanoscale artificial antigen-presenting cells exhibited improved distribution in lymph nodes, and also stimulated T cells and resident antigen-presenting cells.

### Applications of Modified Cell-Derived Matrices

In recent years, the application of cell-derived matrices (CDMs) as biomaterials evolves rapidly. CDMs contains complex biomolecules, resulting in its highly bioactivity and biocompatibility. However, the scarcity of specific addressable functional groups greatly hinders its biological application (Ruff et al., 2017). In 2017, Ruff et al. developed a novel method, for the first time, for overcoming this limitation, by specifically using MGE to incorporate azide groups into the cellular glycoconjugates of CDMs (Ruff et al., 2017). Similarly, Gutmann et al.(Gutmann et al., 2018; Gutmann et al., 2019) successfully modified the ECM of NIH3T3 fibroblasts with azide groups using the glucosamine derivate 2-azidoacetylamino-2-deoxy-(1,3,4,6)-tetra-O-acetyl-d-glucopyranoside (Ac4GlcNAz). Recently, Keller et al. (2020) used an azide-modified ECM to create homogeneous, dense, stable and highly bioactive cell substrates which can be used for bioconjugation. At present, azide groups are widely used for CDMs metabolic modification, mainly because of their small size, ease of incorporation, absence in nature, and ability to selectively react with alkynes in bioorthogonal 1,3-dipolar Huisgen cycloadditions.

## Prospects

In the near future, we envisage the trend of applications of MGE are as follows: 1) MGE will be used in other new disease models, not just in tumor therapy, such as type 1 diabetes (Au et al., 2021); 2) There will be rapid discovery of new precursors for MGE. Lu et al. (2015) labeled cell surface GPIs and GPI-anchored proteins with artificial inositol derivatives, while Wang et al. (2020) labeled NK cells with CD22 ligands using MPB-sia one and BPC-sia 2; 3) As mentioned above, azide groups are wildly used for MGE, the application of other reporter groups (alkynes, alkenes … ) will be explored in the future; 4) The stability and half-life issue of the functional molecules on the cell could be ameliorated for certain applications, due to cell division and protease degradation; 5) Most of the research in this field has been carried out in cell culture and also in animals. Translating MGE from the laboratory into clinical practice is on the way.

## Conclusion

This safe and reversible MGE strategy endows natural cell membrane with additional chemical functionalities, thereby offering unprecedented opportunities for cellular biological functions modulation and novel therapeutics development. This systematic review of recent applications of MGE affirms this approach’s potential in tissue engineering and regenerative medicine. In the future, new chemical reporter groups and bioorthogonal ligation reactions will further expand the development and application of MGE.
